# Advances in antibiotic therapy in the critically ill

**DOI:** 10.1186/s13054-016-1285-6

**Published:** 2016-05-17

**Authors:** Jean-Louis Vincent, Matteo Bassetti, Bruno François, George Karam, Jean Chastre, Antoni Torres, Jason A. Roberts, Fabio S. Taccone, Jordi Rello, Thierry Calandra, Daniel De Backer, Tobias Welte, Massimo Antonelli

**Affiliations:** Department of Intensive Care, Erasme Hospital, Université libre de Bruxelles, 1070 Brussels, Belgium; Infectious Diseases Division, Santa Maria Misericordia University Hospital, 33100 Udine, Italy; Service de Réanimation Polyvalente, CHU de Dupuytren, 87042 Limoges, France; Infectious Disease Section, Louisiana State University School of Medicine, 70112 New Orleans, LA USA; Réanimation Médicale, Groupe Hospitalier Pitié-Salpêtrière, 75013 Paris, France; Department of Pulmonary Medicine, Hospital Clinic of Barcelona, IDIBAPS-Ciberes, 08036 Barcelona, Spain; Burns, Trauma and Critical Care Research Centre, The University of Queensland, Royal Brisbane and Women’s Hospital, 4029 Herston, Brisbane, Australia; Department of Intensive care, CIBERES, Vall d’Hebron University Hospital, Universitat Autonoma de Barcelona, 08035 Barcelona, Spain; Infectious Diseases Service, Centre Hospitalier Universitaire Vaudois, University of Lausanne, 1011 Lausanne, Switzerland; Department of Intensive Care, CHIREC Hospital, Université Libre de Bruxelles, 1420 Braine L’Alleud, Belgium; Department of Respiratory Medicine, Medizinische Hochschule, 30625 Hannover, Germany; Department of Anesthesiology and Intensive Care Medicine, Catholic University of Rome, A. Gemelli University Hospital, Rome, Italy

## Abstract

Infections occur frequently in critically ill patients and their management can be challenging for various reasons, including delayed diagnosis, difficulties identifying causative microorganisms, and the high prevalence of antibiotic-resistant strains. In this review, we briefly discuss the importance of early infection diagnosis, before considering in more detail some of the key issues related to antibiotic management in these patients, including controversies surrounding use of combination or monotherapy, duration of therapy, and de-escalation. Antibiotic pharmacodynamics and pharmacokinetics, notably volumes of distribution and clearance, can be altered by critical illness and can influence dosing regimens. Dosing decisions in different subgroups of patients, e.g., the obese, are also covered. We also briefly consider ventilator-associated pneumonia and the role of inhaled antibiotics. Finally, we mention antibiotics that are currently being developed and show promise for the future.

## Background

Intensive care unit (ICU) patients are particularly likely to have or develop infection, in part because infection is a reason for admission and in part because of immunosuppression associated with critical illness and the large number of invasive devices used in these patients. Correct and adequate antibiotic coverage is essential but can be complex as a result of delayed identification of microorganisms, the impact of critical illness and therapy on pharmacokinetics (PK) and pharmacodynamics (PD) of antibiotics, and the high prevalence of antibiotic-resistant strains.

In this review, we briefly highlight the importance of early infection diagnosis before discussing some of the key issues related to antibiotic management, including problems associated with timing, duration, and dosing. We also briefly consider ventilator-associated pneumonia (VAP), the use of inhaled antibiotics, and new antibiotic and adjunct strategies for the future. We focus on bacterial infections and issues associated with multi-drug resistance will not be covered.

## Diagnosis

The diagnosis of infection in critically ill patients and identification of causative microorganisms and their antibiotic susceptibilities can be a challenge and yet early, appropriate antibiotic therapy is associated with improved outcomes [1], so accurate, rapid diagnosis is important. Typical clinical signs of infection, such as fever or raised white blood cell count, are non-specific and can occur in many other conditions in the critically ill population. Similarly, although many biomarkers, e.g., C-reactive protein and procalcitonin (PCT) to name just two [2], have been suggested to help diagnosis or to rule out infection, none is specific for infection and all can be altered in other conditions that commonly affect ICU patients. Diagnosis of infection still relies largely on culture-based techniques, which can take several days for a positive result to be available. Moreover, in patients already receiving antibiotics, cultures may be negative.

In response to this problem, more rapid microbiological identification methods are being developed, including polymerase chain reaction (PCR) and mass spectrometry with or without electrospray ionization [3–6]. These tests, particularly when associated with an antimicrobial therapy team or pharmacist trained in infectious diseases, may result in shorter times to effective therapy, shorter lengths of hospital stay, and reduced hospital costs [3, 4] and are likely to become more widely used in the near future [7].

## Antibiotic therapy

### Empiric treatment

It is generally accepted that antibiotics should be administered as soon as possible once infection is identified [8], although randomized data to support this notion are lacking in humans for obvious ethical reasons, and most data are from observational studies.

There has been and still is considerable debate regarding the potential benefits of combination versus monotherapy in the empiric management of infection in critically ill patients. Combination therapy has advantages and disadvantages (Table 1). A first potential advantage is in vitro synergy between two drugs resulting in improved bacterial killing. For example, a colistin–glycopeptide (vancomycin or teicoplanin) combination was shown in vitro to be synergistic against multidrug-resistant (MDR) Gram-negative bacteria, especially *Acinetobacter baumannii* [9, 10]. Nevertheless, clinical studies have been unable to demonstrate an effect of synergy on outcomes [11, 12], calling into question the importance of synergy with the potent antibacterial agents used as monotherapy today. Another potential advantage is that combination regimens may provide a greater overall spectrum of activity.

**Table 1:** Some potential advantages and disadvantages of using combination empiric therapy versus monotherapy

Advantages	Disadvantages
Broader coverage that includes non-susceptible strains	Possible antagonism
Anti-bacterial synergy	Possible superinfection
Prevents emergence of resistance	May increase resistance
	Increased toxicity
	Increased costs

One of the most important potential disadvantages of combination therapy is increased drug toxicity, particularly when aminoglycosides are used [13]. Although this increased risk may be acceptable in a critically ill population with a high risk of MDR organisms, it is likely less acceptable in more stable patient populations or where the risk of β-lactam resistance is lower. Risk of superinfection with resistant bacteria or fungal infections represents another potential disadvantage [14]. Another frequently cited disadvantage of combination therapy is increased cost. However, although drug costs will almost certainly be higher with combination therapy, this increased cost may be acceptable if compensated for by shorter hospital stays and improved patient outcomes.

In a cohort of patients with septic shock, combination therapy of a β-lactam with other antibiotics was associated with a decrease in 28-day mortality compared with β-lactam monotherapy [15]. And, in a prospective, multicenter European observational study, combination therapy with macrolides was associated with better outcomes compared with monotherapy in mechanically ventilated patients with community-acquired pneumonia (CAP) [16]. However, not all studies have demonstrated an advantage of combined therapy over monotherapy [17–20]. Importantly, all these studies have compared different antibiotic regimens in different patient populations, making it difficult to generalize the results. In addition, severity of illness can play an important role when comparing mono- and combination therapy. In a meta-regression analysis, Kumar et al. [21] reported that although there was no overall mortality/clinical response benefit with combination therapy for the 50 studies included, when studies were stratified according to baseline mortality risk, combination therapy was consistently associated with benefit in the more severely ill patients. Moreover, the benefits of antibiotic therapy, whether combined or monotherapy, are related to the activity of the chosen antibiotics against the infecting organisms and adequacy has rarely been assessed in these studies.

In current guidelines, combination therapy is suggested for neutropenic patients with sepsis, patients with infections caused by MDR pathogens and patients with severe respiratory infections and septic shock [8, 22]. In general, decisions regarding the use of combination or monotherapy should be made on an individual basis according to the severity of the disease, likely causative microorganism(s), concomitant diseases, and local microbiological and resistance patterns.

### De-escalation?

Decisions regarding empiric antibiotic therapy are based on two approaches: (1) a judgment that the likely agent has “normal antibiotic susceptibility” and can therefore be treated as such with possible need for “escalation” to second-line drugs after microbiological identification; (2) a judgment, based on local microbiology patterns and clinical presentation, that the infecting microorganism may be MDR and should be treated as such, with possible “de-escalation” to a simpler antibiotic regimen after identification and antibiotic susceptibilities of the causative microorganism are known. More frequently the latter approach is used in the ICU to ensure that all possible causative organisms are initially covered. Indeed, only about 30 % of all antibiotics are used for definitive therapy in which the susceptibility patterns for the infection-associated pathogen are known [23, 24]. In many ICUs, more than 50 % of isolates are resistant to at least one antibiotic [25], and broad-spectrum combination empiric therapy may be warranted in these units to ensure that these organisms are adequately covered. Once susceptibilities are confirmed, the spectrum can be reduced (de-escalated) accordingly, although one study reported that de-escalation may actually be feasible in <50 % of cases [26].

Studies have reported conflicting effects on outcomes with de-escalation in various groups of critically ill patients [27–29]. A systematic review of 493 studies concluded that there was not sufficient evidence to determine whether de-escalation of antibiotic agents was effective and safe for adults with sepsis [30]. Nevertheless, within a context of a dedicated “antibiotic stewardship” program, de-escalation should be encouraged, whenever possible, to optimize antibiotic use [31, 32].

### When to stop?

Longer antibiotic courses are associated with MDR pathogen selection and spread, increased risks of toxicity, and higher costs, but courses that are too short risk inadequate bacterial eradication and relapse. Current guidelines advise a 7–10 day course, unless poor prognosis predictors are present (e.g., initial clinical failure, undrainable foci of infection) [8]. Infections caused by *Staphylococcus aureus* or *Pseudomonas aeruginosa* may warrant more prolonged antibiotic courses to avoid treatment failures, early relapses or metastatic complications. CAP, with the exception of *Legionella* pneumonia, should not be treated for more than 8 days in responding patients and invasive abdominal infections may be successfully managed with a 7-day course [22, 33] or even a 4-day course when the source is controlled [34]. A systematic review of 24 studies that compared a shorter (5–7 day) regimen versus a longer (7–21 day) antibiotic course for critically ill patients with various infections identified no differences in terms of clinical cure, microbiological eradication, or survival [35]. Decisions about duration of antibiotic therapy need to be individualized, taking into account different variables regarding the patient (e.g., severity of illness, clinical response), the type of infection (e.g., source control, deep-seated infection [e.g., bone infection], MDR pathogens) and the availability of diagnostic tools (e.g., clinical/laboratory scores, biomarker). An 8-day course will likely be more than sufficient in most ICU patients, and shorter courses may be considered when the source is controlled.

Biomarkers may assist in decisions regarding when to stop antibiotics. Concentrations of PCT, a 116 amino acid peptide, increase during infection and sepsis in correlation with the degree of inflammatory response and the severity of the disease. However, PCT concentrations also increase in some non-septic conditions [36] and remain low in some microbiologically proven bacterial infections, especially when the infectious process remains localized. Nevertheless, PCT concentrations decline quickly when infection is controlled, so that its kinetics during the course of the disease may facilitate decisions to discontinue antibiotics. There is no clear PCT cutoff value to decide when to stop antibiotics, although high values (>1 ng/mL) are strongly suggestive of active bacterial infection. A value <0.5 ng/mL or a decrease >80 % from the initial value may be used as a threshold value to stop antibiotics in stable patients. This approach has been evaluated in several randomized controlled trials (RCTs) [37–40]. In the PRORATA trial [40], which included 621 ICU patients half of whom had septic shock, patients in whom antibiotics were started or stopped according to PCT concentrations had significantly more days without antibiotics than controls (14.3 versus 11.6, *p* < 0.001), without apparent harm.

### Dosing issues

#### Pharmacokinetics/pharmacodynamics

Various PK factors are altered in critically ill patients and can have profound effects on the attainment of adequate antibiotic doses:

##### Target site penetration

Because most infection occurs in tissue interstitial fluid, the antibiotic concentration measured in the plasma is actually often only a surrogate for the true concentration at the site of infection and may over- or underestimate the actual interstitial fluid concentration. In critically ill patients, microvascular failure may impair target site penetration [41].

##### Clearance

Several variables can affect the renal clearance of hydrophilic antibiotics. In the setting of hypoalbuminemia, there is enhanced clearance of highly protein-bound drugs. For patients with high cardiac output and low systemic vascular resistance, as in sepsis, renal clearance of drugs may be augmented by increased renal perfusion, to as much as triple the normal rate [42–44], and may be associated with treatment failure despite the patient being susceptible to the antibiotic.

##### Volume of distribution

Multiple factors have been shown to increase the volume of distribution (Vd) of antibiotics beyond the traditionally accepted fluid extravasation that impacts hydrophilic antibiotics. These include an increase in Vd associated with fluid resuscitation or the physiologic derangements occurring with increased severity of illness [45].

Recently, an enhanced understanding of the PK of antibiotics has developed, largely based on the hydrophilicity of the agents [46]. With hydrophilic agents (e.g., β-lactam antibiotics, aminoglycosides, glycopeptides, lipopeptides), tissue distribution is limited to the extracellular space, and clearance is predominantly via renal mechanisms. By contrast, with lipophilic agents (e.g., fluoroquinolones, glycylcyclines, lincosamides, macrolides, metronidazole, streptogramins, tetracyclines) tissue distribution includes intracellular penetration and hepatic clearance is more common. These variables become important in septic patients because hydrophilic antibiotics require an increased loading dose in the setting of sepsis to ensure therapeutic concentrations are achieved early. With lipophilic agents, an increased loading dose in septic patients is not needed and dose adjustment of these antibiotics is generally only required in the setting of severe hepatic failure [46].

The bacterial killing characteristics of antibiotics are mostly characterized in terms of time-dependent and concentration-dependent killing [47]. With time-dependent antibiotics, such as β-lactams and glycopeptides, maximum bacterial killing occurs when the drug concentration persistently exceeds the minimum inhibitory concentration (MIC) of the pathogen. By contrast, with concentration-dependent antibiotics, such as aminoglycosides and fluoroquinolones, maximum bacterial killing occurs when the peak drug concentration exceeds several times (>8–10) the MIC.

In a prospective multinational point-prevalence study of 361 evaluable patients in which 248 patients were treated for infection, 16 % did not achieve target-free antibiotic concentrations above the MIC. Of these patients, 32 % were less likely to have a positive clinical outcome [48]. These data provide an important glimpse into the relevance of PK/PD issues in the management of critically ill patients, and they challenge healthcare providers managing patients in the ICU setting to move away from the “one dose fits all” strategy that has been traditionally employed in clinical medicine and toward a more personalized antibiotic dosing that is individualized to the physiology of the patient being treated [45]. Importantly too, these changes are unpredictable and measurement of drug concentrations will increasingly be employed to ensure doses are adequate.

#### Doses of β-lactams

β-Lactams are time-dependent antibiotics. Several studies have shown that β-lactam concentrations are inadequate in patients with sepsis compared with non-critically ill patients when standard dosage regimens are administered, particularly when dealing with difficult-to-treat strains such as *P. aeruginosa* [49, 50]. To improve PD target attainment, β-lactams can be administered at increased doses, increased frequency, or by an extended or continuous infusion. Among these options, continuous infusions are often used in critically ill patients and have repeatedly been shown to achieve higher steady-state β-lactam concentrations compared with trough concentrations with standard intermittent regimens [51], although outcome benefits have not been clearly demonstrated [52]. Interestingly, even when given by continuous infusion, β-lactam concentrations can still remain below the MIC for difficult-to-treat pathogens, especially in patients with a high creatinine clearance associated with high renal drug elimination [53]. Importantly, β-lactams have been associated with neurotoxicity and high β-lactam concentrations may be implicated in clinical neurological deterioration [54]. It is important, therefore, to ensure that concentrations remain within therapeutic ranges.

#### Doses of aminoglycosides

To be active, aminoglycosides need to reach peak concentrations at least eight times higher than the MIC for the strain, while low trough concentrations need to be achieved (high concentrations are associated with toxicity). Due to changes in Vd and renal clearance, the PK of aminoglycosides may be altered in sepsis, especially in septic shock, leading to insufficient peak concentrations. Accordingly, aminoglycoside doses have been revisited. For example, the recommended loading dose of amikacin has been increased from 15 to 25 mg/kg and even these doses may not be high enough in some patients. In a multicenter trial in 80 patients, administration of 25 mg/kg of amikacin allowed adequate peak concentrations to be achieved in only 70 % of patients [55]. Similar results were observed in a trial of 146 patients [56] in which the higher the fluid balance the lower the chances of reaching adequate concentrations, highlighting the crucial role of changes in aminoglycoside Vd. Importantly, toxic trough concentrations were uncommon in both trials.

When very high peak concentrations need to be reached, as in patients with MDR pathogens with intermediate susceptibility to aminoglycosides, combination of very high doses of aminoglycosides associated with high-flow (50 ml/kg/min) continuous veno-venous hemofiltration may help achieve adequate peak concentrations while minimizing toxicity and, even more importantly, enable daily administration of the agent (and thus more frequent exposure to its bactericidal effects) [57].

#### Dosing in obese patients

Obesity is associated with different physiological distributions of protein and water-based tissue (e.g., muscle) and lipid-based tissue (e.g., fat) than are present in non-obese patients. These patients tend to also have a higher blood volume and cardiac output than their non-obese counterparts and are believed to have reduced perfusion of peripheral tissues. These factors can lead to changes in Vd and drug clearance that necessitate different drug doses to achieve the same concentrations observed in non-obese patients.

Various metrics have been used to help describe drug behavior in obese patients. In general, for antibiotics primarily eliminated by the kidneys, an accurate description of glomerular filtration rate or creatinine clearance is sufficient for predicting drug clearance in obesity. Many equations, such as the Cockroft-Gault and Modified Diet in Renal Disease equations, do not perform particularly well at extremes of body weight, in which case less common equations, such as the Salazar-Corcoran equation [58], should be substituted where measured urinary collection is not possible.

For aminoglycosides, adjusted body weight is considered the best descriptor of Vd, with clearance varying to a similar extent such that half-life is often the same in obese and non-obese patients [59]. For glycopeptides, total body weight (TBW) is the most accurate descriptor of Vd and clearance changes [60]. For β-lactams, there is a lack of consensus amongst the sparse papers, but lean body weight (LBW) is a plausible descriptor for changes in Vd and drug clearance is well described by creatinine clearance [61]. For fluoroquinolones, the data are not completely clear, but LBW seems an appropriate descriptor of changes in Vd for levofloxacin and either LBW or TBW appear appropriate for ciprofloxacin [62]. For linezolid, there are insufficient data to make strong recommendations for altered dosing in obesity, although differences in PK are considered likely. For daptomycin, TBW has been correlated with changes in Vd and drug clearance [63].

#### Dosing during extracorporeal therapies

Renal replacement therapy (RRT) can be delivered by diffusion (hemodialysis), convection (hemofiltration), or a combination of both (hemodiafiltration). It may be delivered continuously (CRRT) or intermittently. There are several papers on drug dosing during CRRT, but very few in critically ill patients receiving intermittent RRT [64]. A specific issue for intermittent RRT is the inconsistent drug clearance likely to occur during a 24-hour period [65]. Such inconsistent clearance is highly problematic for time-dependent antibiotics where unadjusted dosing in the presence of high drug clearance that alternates with no drug clearance will result in potentially very low and very high concentrations over the course of the day exposing the patient to risks of clinical failure and toxicity.

Drugs that are hydrophilic and usually subject to renal clearance are commonly cleared by dialysis [66]. Large molecules (>1000 Da), like vancomycin, are poorly cleared by hemodialysis, although the availability of high-flux filters has increased the clearance of these drugs somewhat. Smaller molecules, like the β-lactam and aminoglycoside antibiotics, are largely cleared by hemodialysis [66], although this clearance is typically lower than with normal renal function. Protein binding has important effects on drug clearance with highly protein-bound drugs, like teicoplanin, oxacillin and ceftriaxone, having low dialysis clearance because the protein-bound fraction cannot be cleared [66]. Finally, for antibiotics with a larger Vd there is typically less antibiotic in the vascular compartment and so less is available for clearance. This is seen with the quinolones, which have a comparatively larger Vd than the β-lactams or aminoglycosides.

In the absence of clinical PK and dosing data for hemodialysis in critically ill patients, valuable mechanistic insights can be gained from in vitro RRT models. Such experiments have shown that dialysate flow rate is the most important factor associated with hemodialysis clearance of drugs [67]. More data are needed to improve dosing in hemodialysis as the sub-optimal achievement of target concentrations seen in the early phase of therapy in critically ill patients receiving CRRT is likely to also be problematic with intermittent techniques [68].

Antibiotics are commonly required during extracorporeal membrane oxygenation (ECMO); however, few data are available regarding antibiotic PK during ECMO. The major changes in ECMO are increased Vd and decreased drug clearance, although the extent of such changes remains poorly characterized [69]. Antibiotic concentrations may be further altered during ECMO because of the circuit itself (with associated drug sequestration) and/or the associated systemic inflammation (with vasodilation and capillary leak) [70].

The Vd and clearance of meropenem, piperacillin and vancomycin seem to be similar in adult patients undergoing ECMO and in controls [71], suggesting that ECMO may not greatly influence antibiotic PK.

## A special situation: VAP

Although its incidence varies widely according to the population and the criteria used [72], VAP is the leading cause of nosocomial infection in the ICU and a risk factor for increased mortality.

### Diagnosis of VAP

**Fig. 1:**
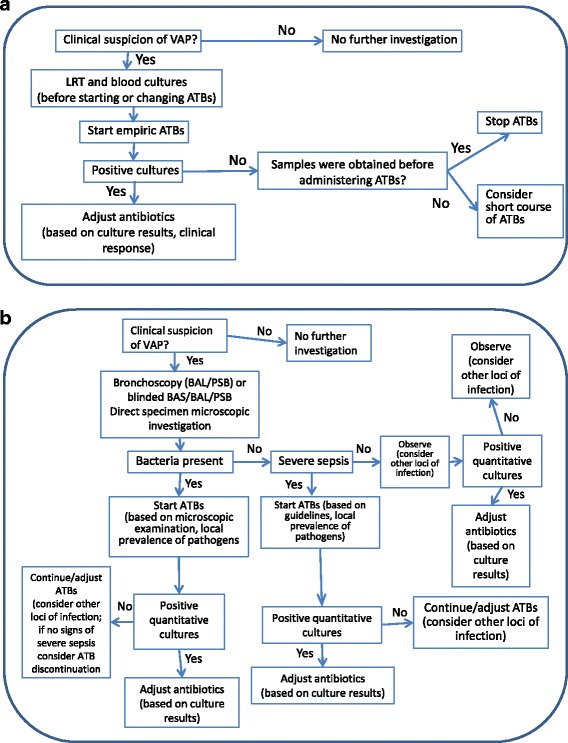
Clinical (**a**) and microbiological (**b**) strategies for diagnosis and management of ventilator-associated pneumonia (*VAP*). *ATB* antibiotic, *BAL* bronchoalveolar lavage, *BAS* bronchial aspirate, *LRT* lower respiratory tract, *PSB* protected specimen brush. Modified from [116] with permission

Diagnosis of VAP is still a difficult clinical issue with two basic diagnostic strategies [73]: clinical and microbiological (Fig. 1). A recent Cochrane review of five randomized studies [74] found no differences in any of the clinical outcomes between these strategies, although an earlier meta-analysis reported that invasive testing was associated with more antibiotic modifications [75]. A recent study [76] proposed that a modified Clinical Pulmonary Infection Score (CPIS), which included lung echography and serum PCT concentrations, would add sensitivity and specificity to the classic CPIS, but this requires further validation. Rapid PCR techniques may help increase the sensitivity and specificity of the clinical suspicion of VAP.

### Should we consider preemptive therapy in VAP?

Respiratory tract colonization precedes VAP in nearly 100 % of cases. Risk factors include prior antibiotics, out-of-hospital intubation, presence of tracheal intubation devices, and duration of intubation. Bacterial biofilm formation on the endotracheal tube may play a key role in colonization. Hospitalized patients tend to become colonized with organisms in the hospital environment within 48 hours. Thus, VAP pathogenesis could be considered as a continuum from ICU admission to confirmed pneumonia through colonization and invasion depending on virulence factors.

Very few trials have tested use of preemptive antibiotics in VAP. Several studies have shown reduced rates of VAP development in patients with ventilator-associated tracheobronchitis (VAT) who received appropriate antibiotic therapy [77–79]. VAT is believed—although this remains controversial— to be an intermediate process between colonization and VAP. In post-cardiac surgery patients at risk of VAT/VAP, Bouza et al. [80] reported a reduction in the VAT/VAP rate in patients who received a 3-day preemptive course of linezolid and meropenem compared with those who did not, but this approach was associated with development of linezolid resistance. In another study, a single dose of ceftriaxone, ertapenem, or levofloxacin within 4 h of endotracheal intubation in comatose patients was associated with reduced early-onset VAP with no increased incidence of infection by multiresistant microorganisms [81]. Clearly further research is needed before any recommendations can be made regarding the use of pre-emptive therapy in VAP.

### A place for nebulized antibiotics in VAP?

For patients receiving mechanical ventilation, aerosolized antibiotics delivered via an efficient system may achieve airway-drug concentrations 100–300-fold higher than the MIC of most bacteria, including MDR pathogens, with reduced systemic toxicity and reduced pressure for selection of resistant organisms [82–84]. In a double-blind, placebo-controlled study in 42 ICU patients requiring prolonged mechanical ventilation who were colonized and/or infected with potentially difficult-to-treat pathogens (e.g., methicillin-resistant *S. aureus* [MRSA] and non-fermenting Gram-negative bacteria), aerosolized antibiotics successfully eradicated the existing MDR organisms and reduced the pressure for new resistance [85].

However, various technical issues need to be addressed. During mechanical ventilation, large droplets (>5 μm) are more likely to be trapped in the circuit, whereas smaller particles (<0.5 μm) are more likely to be expulsed during expiration, so that the size of the particles generated should optimally be between 1 and 3 μm. Particle size depends on the aerosol generator and ventilator settings. On ultrasonic nebulizers, aerosol particle size is inversely proportional to the piezoelectric crystal vibration frequency, and drug output is directly proportional to the amplitude of crystal vibration. On vibrating mesh nebulizers, droplet size is more homogeneous and easier to calibrate [86]. To increase lung deposition, tidal volume has to be set at 500 mL (or more) in adults, with a long inspiratory time (which can be obtained by increasing the I:E ratio) and reduced inspiratory flow. When using a heat/moisture exchanger, it has to be removed during nebulization (and replaced at the end of the aerosol treatment). When using a heated humidifier, it should be switched off during nebulization or the amount of drug should be increased [87].

Several antibiotics have been studied as aerosolized agents; however, how their dosing should be adjusted for optimal efficacy and safety remains unclear. Studies suggest that nebulized aminoglycosides are superior to intravenous administration for providing high tissue concentrations and inducing rapid and potent bacterial killing [88, 89], but renal toxicity may be a concern. In 40 patients with VAP randomized to nebulized or intravenous amikacin and ceftazidime, acquisition of per-treatment antibiotic resistance occurred only in the intravenous group [90]. In a meta-analysis of 12 studies, nebulized antibiotics were associated with improved clinical cure rates in VAP, although this effect did not persist after trial sequential analysis and there was no effect on microbiological cure, lengths of stay, or mortality [91].

In recent years, there has been interest in the use of inhaled colistin (in the form of colistimethate sodium) in VAP because of its known activity against MDR Gram-negative bacteria and the poor penetration of the intravenous form in the lungs [92]. Studies on aerosolized colistimethate as an adjunct to intravenous colistimethate for treating VAP yielded conflicting results [93–96]. A recent meta-analysis of studies suggested that addition of aerosolized colistin in VAP was associated with improved clinical and microbiological response rates but had no effect on mortality [97], but the quality of the evidence was poor.

## New antibiotics in the pipeline

Very few new antibiotics have been developed over the past 10 years but there are some promising agents in the pipeline (Table 2) [98]. Solithromycin, a new macrolide (fluorketolide), effectively kills macrolide-susceptible pathogens, like *Streptococcus pneumoniae*, *Haemophilus influenzae*, and atypical pathogens, and is also effective against macrolide-resistant bacteria. Solithromycin resistance has not yet been identified. In a phase II study in 132 patients with moderate to severe CAP, clinical and microbiological success rates were similar in patients treated with solithromycin (800 mg on day 1, 400 mg from day 2) or levofloxacin (750 mg daily) [99]. Adverse effects, especially diarrhea, were significantly more frequent with levofloxacin (45.6 versus 29.7 %).

**Table 2:** The most important new antibiotic agents in the pipeline

Drug class	Drug name	Development phase	Potential indications
Cephalosporin	GSK-2696266	Phase 1	Bacterial infections
Novel cephalosporin + β-lactamase inhibitor	Ceftolozane + tazobactam	Approved March 2015	Complicated urinary tract infections, complicated intra-abdominal infections, acute pyelonephritis (kidney infection), hospital-acquired bacterial pneumonia/ventilator associated pneumonia
	Ceftaroline + avibactam	Phase 2	Complicated urinary tract infections
	Ceftazidime + avibactam (CAZ-AVI)	Approved 2015	Complicated urinary tract infections, complicated intra-abdominal infections, acute pyelonephritis (kidney infection), hospital-acquired bacterial pneumonia/ventilator-associated bacterial pneumonia
Monobactam + novel β-lactamase inhibitor	Aztreonam + avibactam (ATM-AVI)	Phase 1	Bacterial infections
Carbapenem + novel β-lactamase inhibitor	Carbavance	Phase 1	Complicated urinary tract infections, complicated intra-abdominal infections, hospital-acquired bacterial pneumonia/ventilator-associated bacterial pneumonia, febrile neutropenia
	MK-7655 + imipenem/cilastatin	Phase 2	Complicated urinary tract infections, acute pyelonephritis, complicated intra-abdominal infections
Aminoglycoside	Plazomicin	Phase 3	Bloodstream infections and nosocomial pneumonia caused by carbapenem-resistant Enterobacteriaceae
Fluoroquinolone	WKC 771	Phase 1	Bacterial infections
	WKC 2349 (WCK 771 pro-drug)	Phase 1	Bacterial infections
	Avarofloxacin	Phase 2	Community-acquired bacterial pneumonia, acute bacterial skin and skin structure infections
	Finafloxacin	Phase 2	Complicated urinary tract infections, acute pyelonephritis (kidney infection), acute intra-abdominal infections, acute bacterial skin and skin structure infections
	Nemonoxacin	Phase 2	Community-acquired bacterial pneumonia, diabetic foot infection, acute bacterial skin and skin structure infections
	Zabofloxacin	Phase 2	Community-acquired bacterial pneumonia
	Delafloxacin	Phase 3	Acute bacterial skin and skin structure infections, community-acquired bacterial pneumonia, uncomplicated gonorrhea
Oxazolidinone	Tedizolid	Approved June 2014	Acute bacterial skin and skin structure infections, hospital-acquired bacterial pneumonia/ventilator acquired bacterial pneumonia
	Cadazolid (quinolonyl-oxalidinone)	Phase 3	*Clostridium difficile*-associated diarrhea
	Radezolid	Phase 2	Acute bacterial skin and skin structure infections, community-acquired bacterial pneumonia
	MRX-I	Phase 1	Bacterial infections including community-acquired MRSA and vancomycin-resistant enterococci infections
	LCB01-0371	Phase 1	Bacterial infections
Lipopeptide and glycopeptide	Oritavancin	Approved August 2014	Acute bacterial skin and skin structure infections
Glycopeptide-cephalosporin heterodimer	TD-1607	Phase 1	Serious Gram-positive bacterial infections (acute bacterial skin and skin structure infections, hospital-acquired pneumonia/ventilator-associated pneumonia, bacteremia)
	TD-1792	Phase 2	Acute bacterial skin and skin structure infections, other serious infections caused by Gram-positive bacteria, including hospital-acquired pneumonia/ventilator-associated pneumonia and bacteremia
Lipo-glycopeptide	Dalbavancin	Approved May 2014	Acute bacterial skin and skin structure infections
	Ramoplanin	Phase 2	*Clostridium difficile*-associated diarrhea
Lipopeptide	Surotomycin	Phase 3	*Clostridium difficile*-associated diarrhea
Macrolide			
Ketolide	Solithromycin	Phase 3	Community-acquired bacterial pneumonia, uncomplicated urogenital gonorrhea
LptD inhibitor	POL7080	Phase 2	Ventilator-associated bacterial pneumonia, low respiratory infections
Tetracycline	Omadacycline	Phase 2	Community-acquired bacterial pneumonia, acute bacterial skin and skin structure infections, complicated urinary tract infections
	Eravacycline	Phase 3	Complicated intra-abdominal infections, complicated urinary tract infections, hospital-acquired bacterial pneumonia
Monosulfactam	BAL30072	Phase 1	Multidrug-resistant Gram-negative bacterial infections
Fabl inhibitor	Debio 1452	Phase 2	Acute bacterial skin and skin structure infections
	Debio 1450 (Debio 1452 pro-drug)	Phase 1	Bacterial infections
	CG-400549	Phase 2	Acute bacterial skin and skin structure infections; osteomyelitis
LpxC inhibitor	ACHN-975	Phase 1	Bacterial infections
DNA gyrase inhibitor	AZD0914	Phase 1	Uncomplicated gonorrhea
Methionyl-tRNA synthetase (MetRS) inhibitor	CRS-3123	Phase 1	*C. difficile* infection
Peptide deformylase inhibitor	GSK-1322322	Phase 2	Acute bacterial skin and skin structure infections
Type 2 topoisomerase inhibitor	GSK-2140944	Phase 2	Respiratory tract infections, acute bacterial skin and skin structure infections
Bicyclolide	EDP-788	Phase 1	Bacterial infections
Pleuromutilin	Lefamulin (BC-3781)	Phase 2	Acute bacterial skin and skin structure infections, community-acquired bacterial pneumonia
Elongation factor inhibitor	LFF571	Phase 2	*C. difficile*-associated diarrhea
Fusidane	Taksta (fusidic acid)	Phase 2	Prosthetic joint infections
Defensin-mimetic	Brilacidin	Phase 2	Acute bacterial skin and skin structure infections
	SMT19969	Phase 2	*C. difficile*-associated diarrhea

Adapted from [98] with permission

Omadacycline (an aminomethylcycline) and eravacycline (a fluorocycline) are developed from the tetracyclines and have now entered phase II clinical trials. Omadacycline is available for intravenous and oral therapy. It is effective against a large number of sensitive but also resistant Gram-positive pathogens (including vancomycin-resistant enterococci and MRSA) and against some Gram-positive pathogens, such as *H. influenzae*, *Klebsiella*, and *Escherichia coli* [100]. Eravacycline is also active against resistant Gram-negative pathogens but not against *P. aeruginosa* or *Burkholderia.*

Fifth-generation cephalosporins with MRSA activity (ceftaroline, which does not have *Pseudomonas* activity, and the *Pseudomonas*-active ceftobiprole) are available in a number of countries, but have not been used extensively (because of a lack of data) in critically ill patients. In a recent RCT, ceftobiprole (3 × 500 mg) was compared with the combination of ceftazidime (3 × 2 g) and linezolid (2 × 600 mg) in 781 patients with nosocomial pneumonia, including 210 with VAP [101]. Clinical cure rates overall were around 50 % in both groups, but ceftobiprole performed less well in VAP (23.1 versus 36.8 % cure rate). Ceftolozane/tazobactam is a new cephalosporin that differs from ceftazidime by a modification of the side chain in the third position of the cephem nucleus, allowing increased antipseudomonal activity and activity against some extended spectrum β-lactamase-producing strains. In a recent study in patients with complicated intra-abdominal infection, ceftolozane/tazobactam (3 × 1.5 g) plus metronidazole was non-inferior to meropenem (3 × 1 g) [102]. In another large trial, treatment with ceftolozane/tazobactam was also associated with better responses compared with high-dose levofloxacin in patients with complicated lower urinary tract infections or pyelonephritis [103].

Tedizolid is a new oxazolidinone that is more bactericidal than the currently used linezolid [104]. Although differences in clinical response rates have not been very significant, the rate of adverse events seems to be somewhat lower with tedizolid [105].

Avibactam is a new β-lactamase inhibitor active against a large number of extended spectrum β-lactamases, including class A, some class C, and some class D β-lactamases. It is not active against the metallo-β-lactamases, but is active against *Klebsiella pneumoniae* carbapenemases. Ceftazidime/avibactam combination (plus metronidazole) therapy has been tested against meropenem or imipenem in two RCTs in patients with intra-abdominal and urogenital infection. Non-inferiority of the new combination was demonstrated in both studies [106, 107]. A study comparing ceftazidime/avibactam with meropenem in patients with nosocomial pneumonia is ongoing (ClincialTrials.gov identifier NCT01808092).

Two other new β-lactamase inhibitors are now being studied in phase III trials in patients with MDR enterobacteriaceae, including carbapenem-resistant strains. The boronate β-lactam inhibitor, RPX7007, combined with the new carbapenem, biapenem (RPX2003), demonstrated high bactericidal activity against carbapenem-resistant enterobacteriaceae [108]. MK-7655 (relebactam), in combination with imipenem/cilastatin, covers MDR enterobacteriaceae (with the exception of those producing metallo-carbapenemases), but in addition relebactam augments the activity of imipenem against *P. aeruginosa* in general and especially against OprD mutants of this pathogen [109].

## Non-antibiotic, adjunctive therapies

We are facing increasing incidences of bacterial resistance for both community and nosocomial infections and there is a need for alternative, non-antibiotic, adjunctive therapeutic options to decrease antibiotic pressure. In the ICU, the most problematic microorganisms to treat remain *P. aeruginosa* and *S. aureus* because of their resistance profile and *Clostridium difficile* because of its tendency to cause relapse or recurrence.

The only effective adjunctive therapy for *C. difficile* infections appears to be toxin-neutralizing antibodies that target both toxin A and B [110]. Fecal flora reconstitution by fecal transplantation has also been shown to prevent recurrent infections for up to 1 year [111]. Moreover, *C. difficile* infection recurrence rates decreased threefold when oligofructose prebiotics or toxin-neutralizing antibodies were added to standard antibiotics. Recently, spores of *C. difficile* given by mouth were shown to be effective in stopping repeated bouts of *C. difficile* infection, which occur in 25–30 % of patients who suffer an initial episode of diarrhea or colitis [112].

Monoclonal antibodies are probably the most promising adjunctive option for treating *P. aeruginosa*. Repeated doses of a monoclonal antibody targeting *P. aeruginosa* serotype O11 as an adjunctive therapy to antibiotics in *P. aeruginosa* hospital-acquired pneumonia and VAP were associated with a significant resolution rate without immunogenicity [113].

Human monoclonal antibodies are also being developed that specifically bind and neutralize the alpha-toxin of *S. aureus*, for adjunctive therapy in VAP. In a mouse sepsis model, treated animals had a significant reduction in mortality [114] and in a mouse pneumonia model, treatment protected against both methicillin-susceptible *S. aureus* and MRSA strains (unpublished data). Clinical trials are ongoing in patients with VAP.

Some very innovative adjunctive approaches may also be beneficial in severe infections in the ICU. Pore-forming toxins (PFTs) induce lysis of host target cells by forming pores that disrupt the plasma and can cause serious complications associated with high mortality rates. About 30 % of cytotoxic bacterial proteins are PFTs, making them the largest category of virulence factors. Capturing bacterial PFTs with liposomes by mimicking membrane domains thus appears a promising approach, although it is still in pre-clinical development [115].

## Conclusion

Management of infection in critically ill patients is an evolving challenge, in part because of the ever-present threat of MDR strains. Alterations in PK/PD parameters in critically ill patients can complicate dosing issues, yet adequate antibiotic treatment is crucial to optimize survival rates. Therapeutic drug monitoring is likely to be more widely used in the future. Antibiotic choices and durations need to be individualized for each patient according to specific patient characteristics, disease severity, likely infecting organisms, and local resistance patterns. Although more responsible antibiotic prescribing may help reduce antibiotic pressure and development of antibiotic resistance, research needs to continue to try and identify new antibiotics and adjunctive therapies.

## Abbreviations

CAP: Community-acquired pneumonia
CPIS: Clinical pulmonary infection score
CRRT: Continuous renal replacement therapy
ECMO: Extracorporeal membrane oxygenation
ICU: Intensive care unit
LBW: Lean body weight
MDR: Multi-drug resistant
MIC: Minimum inhibitory concentration
MRSA: Methicillin-resistant *Staphylococcus aureus*
PCR: Polymerase chain reaction
PCT: Procalcitonin
PD: Phamacodynamics
PFT: Pore-forming toxins
PK: Phamacokinetics
RCT: Randomized controlled trial
RRT: Renal replacement therapy
TBW: Total body weight
VAP: Ventilator-associated pneumonia
Vd: Volume of distribution
